# Systematic Review of Patient-Reported Outcomes and Complications of Pedicled Latissimus Flap Breast Reconstruction

**DOI:** 10.1055/a-2045-8122

**Published:** 2023-08-02

**Authors:** Emanuela C. Peshel, Claire M. McNary, Catherine Barkach, Elizabeth M. Boudiab, Daniella Vega, Farid Nossoni, Kongkrit Chaiyasate, Jeremy M. Powers

**Affiliations:** 1 Division of General Surgery, Beaumont Health System, Royal Oak, Michigan; 2 Department of Surgery, Oakland University William Beaumont School of Medicine, Auburn Hills, Michigan; 3 Division of Plastic and Reconstructive Surgery, Beaumont Health System, Royal Oak, Michigan; 4 Division of Plastic and Reconstructive Surgery, Department of Surgery, Quillen College of Medicine, East Tennessee State University, Johnson City, Tennessee

**Keywords:** breast reconstruction, latissimus dorsi flap, BREAST-Q, patient satisfaction

## Abstract

The latissimus dorsi (LD) flap is a reliable option for breast reconstruction. This is particularly true in patients with contraindications to abdominally based autologous breast reconstruction. A systematic review of patient satisfaction and health related quality of life following LD breast reconstruction using the BREAST-Q survey was conducted. The scope of the review was to determine the degree of patient satisfaction following the procedure and to examine how patient satisfaction from the pedicled LD flap compares to other breast reconstructive procedures. A literature search on BREAST-Q in LD flap reconstruction was performed. Only articles written in English and in published peer-reviewed journals were included. Studies with less than 20 patients in their sample and those with a follow-up period of less than 1 year were excluded. Five articles representing 331 patients were reviewed, including one case–control study and four retrospective cohort studies. Level of evidence was either III (4) or IV (1). The average age was 53 with average body mass index of 25. Most reconstructions were delayed (67%) and unilateral (88%), and most patients required radiation (79%). The average length of follow-up was 36 months, and the response rate was 75%. Overall, patients who underwent LD flap reconstruction reported favorable outcomes in satisfaction domains and quality of life domains with few complications. A meta-analysis also demonstrated higher satisfaction in LD flap without implants compared with LD flap with implants. Patient-reported outcomes following LD breast reconstruction compare favorably with other techniques of breast reconstruction.

## Introduction

The pedicled latissimus dorsi (LD) flap has reemerged as a reliable option with minimal complications in autologous breast reconstruction. 1 2 The indications, techniques, and complications of the LD flap have been well-described in the available literature; however, there is a paucity of patient-reported outcome measures that illustrate the patient's perspective following LD flap reconstruction. 3 4 BREAST-Q was first introduced in 2009 and is the only validated outcome measure that quantifies health-related quality of life and patient satisfaction following breast reconstructive surgery. 5 6 BREAST-Q is a valuable tool for evaluating surgical outcomes from the patient's perspective and has become rapidly accepted by clinicians. 4 7

The purpose of this study is to conduct a systematic review of the relevant literature on patient-reported outcomes using the BREAST-Q survey following pedicled LD flap breast reconstruction. This will help quantify the degree of postoperative patient satisfaction following LD flap breast reconstruction and compare the technique to other breast reconstruction procedures. The outcomes from this can be utilized by surgeons to better counsel patients on breast reconstruction options and postoperative expectations.

## Methods

A systematic review was performed to identify peer-reviewed publications evaluating patient-reported outcomes using BREAST-Q following traditional LD flap reconstruction. The protocol was registered with the International Prospective Register of Systematic Reviews (PROSPERO, identification number CRD42021248616). This study did not require an ethics review as it summarized findings from existing publications rather than collect primary data.

### Methodology and Article Selection

This review was conducted using the Preferred Reporting Items for Systematic Reviews and Meta-Analyses (PRISMA) guidelines. 8 PubMed, Embase, Cochrane Library, Scopus, Web of Science, and Google Scholar were searched on December 19, 2020 using the terms (“latissimus flap” OR “pedicled latissimus flap”) AND (“breast reconstruct*” OR “mammaplasty”[Mesh] OR “mammaplast*”). Duplicates were removed. Titles and abstracts were screened for relevance by two independent reviewers (C.M. and J.P.). The relevant titles were retrieved and full-text articles were assessed for eligibility by four reviewers (C.M., E.B., C.B., and J.P.). A standardized quality assessment was applied to all articles using the levels of evidence rating scale as described by the American Society of Plastic Surgeons. 9 10

### Inclusion and Exclusion Criteria

The inclusion criteria were formatted based on the PICOS tool, which stands for Population, Intervention, Comparison, Outcomes, and Study type ( Table 1 ). 11 12 The study population included adult women receiving oncological breast reconstruction using the traditional LD flap. Studies with and without comparison groups were included. All studies utilized the BREAST-Q for patient-recorded outcomes. Studies utilizing a nonstandardized version of BREAST-Q or a modified technique of the LD flap were excluded.

**Table 1:** Inclusion and exclusion criteria

Inclusion criteria	Exclusion criteria
• Population: adult women receiving oncological breast reconstruction • Intervention: LD flap breast reconstruction • Comparison: studies with and without control/comparison groups • Outcome: ▪ Primary : standardized BREAST-Q scores ▪ Secondary : patient demographics, surgical technique, radiation, chemotherapy • Study type: any study design with at least 20 patients in sample size and 1 year follow-up time	• Nonstandardized variations of the BREAST-Q and/or numerical Rasch scores unavailable • Modifications of the traditional LD flap technique • Not a peer-reviewed publication (i.e., abstract only) • Level V evidence (i.e., discussion, letters to the editor, review articles) • Full-text unavailable in English

Abbreviation: LD, latissimus dorsi.

Studies were included only if they were published in the English language in peer-reviewed journals and consisted of level IV evidence and higher as described by Chung et al. 9 Meta-analysis, systematic reviews, expert opinion, case reports, conference abstracts, and book chapters were excluded. Only studies with at least 20 patients and a 1-year follow-up time were included in an effort to capture adequately powered studies, reduce the effects of selection and publication bias, and identify long-term patient satisfaction and complications.

### Data Extraction

Articles that met the inclusion criteria were further analyzed for their utilization of the BREAST-Q score. The primary outcome of interest was the numeric BREAST-Q scores, which is scored from 0 (worst) to 100 (best). 4 5 6 The postoperative reconstruction module of the BREAST-Q is composed of two themes: health-related quality of life and patient satisfaction. Each theme addresses a set of specific domains outlined below.

The health-related quality of life domain includes the following:

- Psychosocial well-being.
- Sexual well-being.
- Physical well-being.

The patient satisfaction domain includes the following:

- Satisfaction with breasts.
- Satisfaction with nipples.
- Satisfaction with abdomen.
- Satisfaction with back.
- Satisfaction with care.
- Satisfaction with outcome.

The secondary outcomes of interest included patient demographics, oncological adjuncts to treatment (i.e., chemotherapy, radiation, and axillary node clearance), and surgical complications. The following information was extracted: country of origin, study aim, study design, study year, sample size, average length of follow-up, patient characteristics (age and body mass index [BMI]), surgical technique, oncological treatments (type of resection, chemotherapy, radiation, and axillary clearance), timing of reconstruction, BREAST-Q module response rates, timing of BREAST-Q, surgical complications, and key findings of the study.

### Data Synthesis and Analysis

Continuous variables, such as age and BMI, were reported via standard summary statistics. The meta-analysis was made with two studies comparing LD flap with and without implant. 13 All statistical analyses were conducted with Review Manager (RevMan, version 5.4.1, Cochrane Collaboration, Copenhagen, Denmark). 14 Continuous outcomes were analyzed as mean differences using the inverse variance method. Heterogeneity test was applied for the included articles. Fixed effects models were used.

## Results

**Fig. 1:**
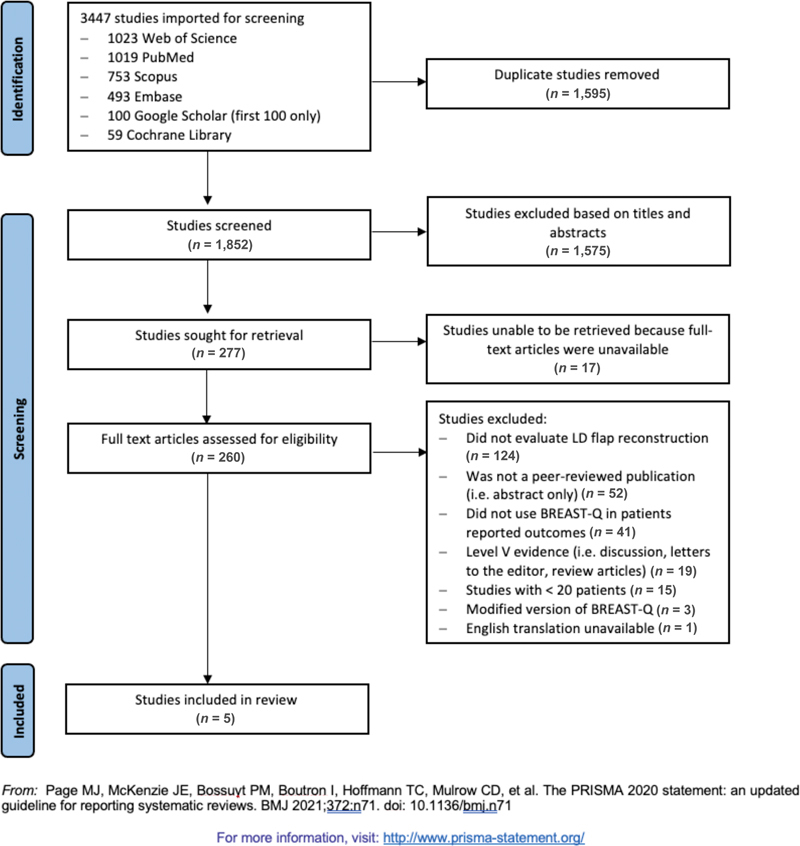
Preferred Reporting Items for Systematic Reviews and Meta-Analyses (PRISMA) flow diagram.

The database search resulted in 1,852 unique articles. After screening the title and abstract, 277 articles were deemed eligible for full-text review. A total of five studies met eligibility criteria and were included for data extraction and analysis ( Fig. 1 ). 13 15 16 17 18 Four studies consisted of level-III evidence and one consisted of level IV evidence per the levels of evidence rating scale as described by the American Society of Plastic Surgeons ( Table 2 ). 13 15 16 17 18 One study was a case-control design, and the remaining were retrospective cohort studies. 13 15 16 17 18 All studies were published between 2018 and 2019. None of the studies received funding.

**Table 2:** Description of included studies

Author, year	Country	Study design	LOE	Study population	Key findings
Koh et al 2018 17	Australia	Case–control	III	LD flap vs. no reconstruction following total mastectomy	Following total mastectomy, patients who received LD flap reconstruction had higher mean BREAST-Q scores compared with patients who did not receive any reconstruction
Leuzzi et al 2019 13	France, Italy	R-Cohort	III	LD flap with implant vs. LD flap without implant	Patients who received LD flap with implant experienced a higher rate of complications and lower BREAST-Q scores within the “satisfaction with breasts” domain compared with patients who received LD flap without implant. Patients who did not have implants with their LD flap did receive a variable number of lipofilling procedures
Menez et al 2018 16	France, Italy	R-Cohort	III	DIEP flap vs. LD flap with implant (LDI) vs. LD flap without implant (ALD)	Patients who underwent immediate breast reconstruction as opposed to delayed breast reconstruction reported statistically higher BREAST-Q scores for the sexual well-being domain. The BREAST-Q scores for the psychosocial well-being domain increased as the age of the patient increased. There were no significant differences in BREAST-Q scores when comparing the three autologous breast reconstruction techniques: DIEP, LDI, and ALD
Rindom et al 2018 18	Denmark, Norway	R-Cohort	III	Delayed LD flap vs. TDAP flap	The LD flap cohort reported significantly lower BREAST-Q scores for the “physical well-being (chest)” domain, with a sub-analysis of the raw scores indicating specifically more frequent pain in the upper back and shoulder. There were no significant differences between the TDAP flap cohort and LD flap cohort in the following BREAST-Q domains: “reconstructed breast,” “overall outcome,” “psychosocial well-being,” “sexual well-being,” and “satisfaction with nipples”
Cattelani et al 2019 15	Italy	R-Cohort	IV	LD flap in salvage mastectomy	Patients who received LD flap in salvage mastectomy reported favorable BREAST-Q scores

Abbreviations: ALD, autologous latissimus dorsi; DIEP, deep inferior epigastric perforator; LD, latissimus dorsi; LDI, latissimus dorsi with implant; LOE, level of evidence; R-Cohort, retrospective cohort; TDAP, thoracodorsal artery perforator flap.

### Patients and Demographics

There were 331 patients represented by the studies reviewed ( Tables 3 and 4 ). The average age was 52.7 years (SD: 2.0) and the average BMI was 25.4 kg/m ^2^ (SD: 0.6). The average length of follow-up was 36.0 months (SD: 11.5). Three-hundred and seventy-two breast reconstructions were performed, 290 (88%) were unilateral, and 41 (12%) were bilateral.

**Table 3:** Patient demographics

Author (year)	Number of patients	Number of reconstructions	Average age (y)	BMI (kg/m ^2^ )	Length of follow-up (mo)	Laterality		Timing			Radiation	Chemotherapy	Axillary
						Unilateral	Bilateral	Immediate	Delayed	Not reported			
Koh et al (2018) 17	100	136	51.0	NR	49.1	64	36	16	44	40	NR	NR	None: 12 SLNBx: 20 ALND: 43 NR: 25
Leuzzi et al (2019) 13	90	95	53.3	26.0	22.3	85	5	4	91	0	Neoadjuvant: 63	Neoadjuvant: 71	NR
Menez et al (2018) 16	58	58	51.7	NR	45	58	0	0	0	58	NR	NR	NR
Rindom et al (2018) 18	24	24	51.0	24.9	33.7	24	0	0	24	0	Adjuvant: 15	Adjuvant: 16	SLNBx: 7 ALND: 15
Cattelani et al (2019) 15	59	59	56.4	24.7	26.7	59	0	59	0	0	Neoadjuvant: 59	Neoadjuvant: 12 Adjuvant: 8	None: 13 SLNBx 39 ALND: 7

Abbreviations: BMI, body mass index; NR, not reported.

**Table 4:** Summary of patient demographics

Study characteristics	*n* ( *N* )	
Total number of patients	5 (331 patients)	
Total number of breasts reconstructed	5 (372)	
Total number of breasts reconstructed with implants	4 (184)	
Patient characteristics	*n* ( *N* )	Mean (SD)
Age (y)	5 (331)	52.7 (2.0)
BMI (kg/m ^2^ )	3 (173)	25.4 (0.6)
Length of follow-up (mo)	5 (331)	36.0 (11.5)
Reconstruction characteristics	*n* ( *N* )	*N* (%)
Laterality	5 (331)	
Unilateral		290 (88%)
Bilateral		41 (12%)
Timing	4 (238)	
Immediate		79 (33%)
Delayed		159 (67%)
History of radiation	3 (173)	137 (79%)

Abbreviations: BMI, body mass index; *N* , number of breasts; *n* , number of studies; SD, standard deviation.

The timing of reconstruction, rates of radiation, chemotherapy, axillary clearance, and complications were inconsistently reported. Timing was reported in 238 (72%) patients, of which 79 (33%) reconstructions were immediate and 159 (67%) were delayed. Rates of radiation and chemotherapy therapy were reported in three studies, which represented 173 (52%) patients. 13 15 18 One-hundred and twenty-two (71%) of the 173 patients with a history of irradiation had reconstructions performed on irradiated tissue. Seventy-three patients required neoadjuvant chemotherapy, while 24 patients required adjuvant chemotherapy. Axillary clearance was reported in three studies representing 183 patients. 15 17 18 Sentinel lymph node biopsy was required in 66 patients, while axillary lymph node dissection was required in 65 patients.

### Complications

Complications were reported in two studies representing 149 (45%) patients ( Table 5 ). 13 15 There were 31 complications, resulting in an overall complicate rate of 21%. Seroma was the most reported complication, occurring in 13 (9%) patients. Flap necrosis or partial loss was reported in six (4%) patients. Of the 149 patients with complications, 112 (75%) received implants. Implant-based complications requiring implant removal occurred in 13 of these patients, corresponding to an implant extraction rate of 12%.

**Table 5:** Reported complications

Author (year)	Total	Seroma	Hematoma	Infection	Flap necrosis or partial loss	Implant-based complications requiring removal
Koh et al (2018) 17	NR	NR	NR	NR	NR	NR
Leuzzi et al (2019) 13	16	4	5	1	1	10 of 53
Menez et al (2018) 16	NR	NR	NR	NR	NR	NR
Rindom et al (2018) 18	NR	NR	NR	NR	NR	NR
Cattelani et al (2019) 15	15	9	0	1	5	2 of 59

Abbreviation: NR, not reported.

### BREAST-Q

The most frequently reported domains of the BREAST-Q were satisfaction with breasts, satisfaction with well-being, sexual well-being, and physical well-being (chest) ( Table 6 ). The average response rate to all BREAST-Q domains was 75%. In the satisfaction with breast domain, the average response rate was 79% and the mean score was 67.0 (SD: 7.6). In the satisfaction with outcome domain, the average response rate was 80% and the mean score was 76.6 (SD: 4.7). In the psychosocial well-being domain, the average response rate was 79% and the mean score was 76.1 (SD: 7.9). In the sexual well-being domain, the average response rate was 66% and the mean score was 62.5 (SD: 6.9). In the physical well-being (chest) domain, the average response rate was 72% and the mean score was 75.5 (SD: 9.6).

**Table 6:** Summary of BREAST-Q results

	Patients ( *n* )	Response rate (%)	Mean (SD)
Satisfaction with breasts	331	79	67.0 (7.6)
Satisfaction with outcome	172	80	76.6 (4.7)
Psychosocial well-being	331	79	76.1 (7.9)
Sexual well-being	331	66	62.5 (6.9)
Physical well-being (chest)	331	72	75.5 (9.6)

Abbreviation: SD, standard deviation.

### Meta-analysis

**Fig. 2:**
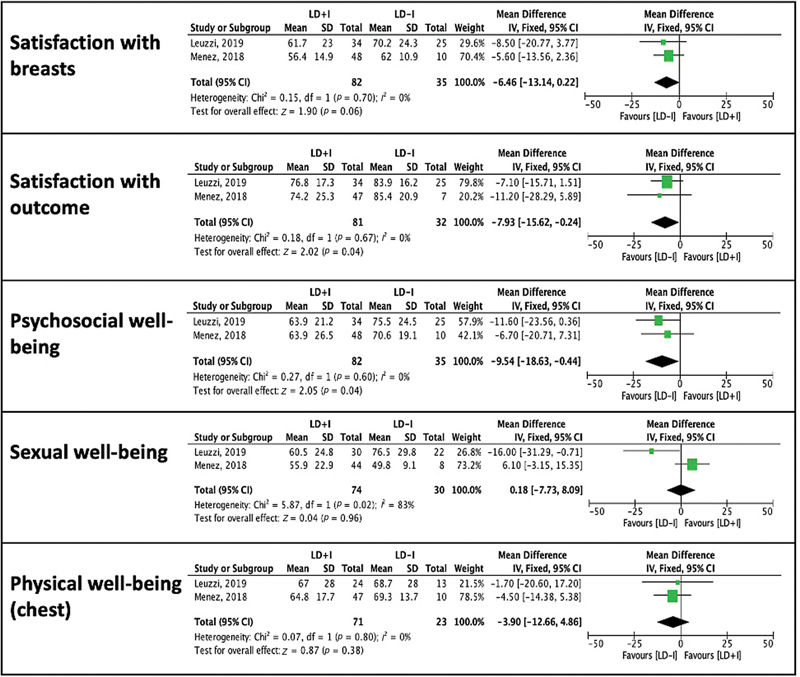
BREAST-Q meta-analysis of LD + I versus LD-I. CI, confidence interval; LD, latissimus dorsi; SD, standard deviation.

A meta-analysis was conducted using two studies. 13 16 There was evidence of clinical and methodical homogeneity allowing for pooling of data, except for the “sexual well-being” domain ( Fig. 2 ). Head-to-head comparison of BREAST-Q scores demonstrated higher scores in patients who underwent LD without implant versus patients who received LD with implant, and this reached statistical significance in satisfaction with outcome and psychosocial well-being domains (mean difference -7.93 [95% confidence interval, CI: −15.62, −0.24], *p* = 0.04 and −9.54 [95% CI: −18.63, −0.44], *p* = 0.04, respectively). Due to the small sample size and the risk of falsely obtaining effects and interactions, no further subgroup analysis was performed.

## Discussion

### BREAST-Q

This review highlights the favorable patient-reported outcomes using BREAST-Q scores ( Table 6 ). Overall, the average BREAST-Q scores reported by the studies included in this review correlated with better patient-reported postoperative satisfaction when compared with prior validation studies. 5 Notably, BREAST-Q scores were higher in patients undergoing LD flap versus no reconstruction and in patients undergoing immediate versus delayed reconstruction. 16 17 Patients who underwent LD flap reconstruction had similar BREAST-Q scores when compared with other autologous reconstruction techniques. 15 18 This is consistent with a large survey study that reported similar BREAST-Q scores between the LD flap and other types of reconstruction. 19

The meta-analysis conducted in this review compared BREAST-Q scores in patients who underwent LD flap reconstruction with and without implants ( Fig. 2 ). Two studies originating from hospital systems located in France were included. 13 16 Patients who did not receive implants with their LD flap had higher scores overall; satisfaction with outcome and psychosocial well-being domains reached statistical significance ( *p* = 0.04 and 0.04, respectively). This is consistent with previous studies that reported higher patient-reported outcomes with autologous breast reconstruction when compared with implant-based reconstruction. 4 19

### Reported Complications

This review also affirms the reliability and safety of the LD flap in oncologic breast reconstruction. Although abdominal-based breast reconstruction has become more common, the LD flap continues to have advantages for patients with a history of chemotherapy and radiation. 13 15 18 20 21 The technique can be adapted for concurrent sentinel lymph node biopsy, axillary lymph node dissection, and axillary clearance or for immediate reconstruction. 15 17 18 A recent systematic review by Fischer et al found that in patients with previously irradiated tissue, the LD flap had a clinically significant decrease in device loss, infection, and reoperation compared with implants alone, though patient-reported outcomes were not evaluated. 3

Despite the known advantages of the LD flap, the main reported disadvantages include donor site morbidity, the inability to provide adequate volume, and loss of implant. 13 16 22 Although agonistic muscles are believed to compensate for the loss of the LD after flap harvest, there may be a negative effect on the overall function of the shoulder, leading to pain or reduced range of motion. 18 Using the Constant Shoulder Scale, Rindom et al reported more frequent pain in the upper back and shoulder in patients with LD flap reconstruction when compared with the thoracodorsal artery perforator flap. 18 Conversely, using the Disability of the Arm Shoulder, and Hand Questionnaire (DASH), Cattelani et al reported no disability in 42 of 59 patients and minimal disability in 17 of 59 patients. 15 In a systematic review and meta-analysis on shoulder function after LD flap reconstruction using the DASH questionnaire, Steffenson et al found that range of motion was most impaired 3 months after LD flap reconstruction, while the overall limitations in function were minimal. 22

Two of the studies included in the review reported complications; the most common complications were seroma, hematoma, and flap or implant loss ( Table 5 ). 13 15 The majority of complications reported by Cattelani et al were within the immediate postoperative period and resolved with conservative measures such as aspiration of seroma or local wound care for superficial necrosis. Only 2 of 59 patients required implant removal. 15 Leuzzi et al reported a higher rate of implant-based complications with 10 of 53 patients requiring removal. 13 The complications in implant-based reconstruction were attributed to the use of implants; no difference was found in other factors that may influence healing, such as radiation. 13

The use of implants in LD flap reconstruction brings focus to the most significant disadvantage of LD flap reconstruction: lack of volume. The lack of volume is mitigated with either an implant or lipofilling procedures. 13 16 In the meta-analysis, patients who had LD flap reconstruction without implants were more likely to require lipofilling procedures to obtain the desired volume. 13 16 Leuzzi et al reported that 80% of patients who had LD flap reconstruction without implants required a lipofilling procedure, as opposed to 50% of patients with implants. 13 Furthermore, Menez et al reported that patients who had LD flap reconstruction without implants required a mean of 1.13 additional lipofilling operations, in comparison to patients with implants, who required a mean of 0.64 additional lipofilling operations. 16 Despite the need for additional lipofilling procedures, patients without implants had higher BREAST-Q scores. 13 16

## Limitations

Limitations of this systematic review and meta-analysis include small sample size, few comparison groups, and heterogeneity within an individual domain. Of the full-text articles assessed for eligibility, 16% (41/260) were excluded because they did not use BREAST-Q to assess patient-reported outcomes ( Fig. 1 ). This significantly reduced the sample size and comparison groups included in the study. This was further reduced by studies that did not contain long-term follow-up of greater than 1 year, which was felt to be imperative in assessing patient-directed outcome measures.

## Future Considerations

Future studies evaluating patient-reported outcomes after LD flap breast reconstruction should utilize the BREAST-Q reconstruction module in addition to the LD flap-specific BREAST-Q scales (satisfaction with back appearance, satisfaction with shoulder and back function). Patients should be assessed in the immediate postoperative period and long-term to capture outcomes years after reconstruction. Additional opportunities for further research will involve applying the standardized BREAST-Q, both preoperatively and postoperatively, to large sample sizes. Doing so will amplify the patient-reported experience and provide surgeons with insight to tailor breast reconstruction techniques to the individual breast cancer patient.

## Conclusion

This systematic review and meta-analysis on patient-reported outcomes using the BREAST-Q survey highlight the favorable outcomes of LD flap breast reconstruction. Despite the need for more lipofilling procedures, patients who undergo immediate LD flap reconstruction without implants report the best outcomes. 13 16 The literature demonstrates that the LD flap continues to be a safe and reliable technique for oncological breast reconstruction. Knowledge of patient-reported outcomes using standardized measures that demonstrate strong validity and reliability, such as the BREAST-Q, will allow surgeons to better counsel patients regarding postoperative expectations.
